# Extrapulmonary tuberculosis in Morocco: A systematic review of observational studies

**DOI:** 10.1590/0037-8682-0066-2024

**Published:** 2025-03-17

**Authors:** Mohamed Dellal, Sanaa Batoui, Ahmed Chetoui, Mohamed Kanouf, Touria Fatihi, Khalid Habbari

**Affiliations:** 1 University Sultan Moulay Slimane, Faculty of Sciences and Techniques, Béni Mellal, Morocco.; 2 Regional Hospital Centre, Béni Mellal, Morocco.

**Keywords:** Extrapulmonary tuberculosis, observational studies, prevalence, risk factors, treatment outcomes, systematic review

## Abstract

**Background:**

Tuberculosis remains a major global health concern and a leading cause of mortality. In Morocco, various forms of extrapulmonary tuberculosis are prevalent. This systematic review examines research findings on the prevalence, risk factors, and treatment outcomes of extrapulmonary tuberculosis in Morocco.

**Methods:**

We conducted searches for observational studies on extrapulmonary tuberculosis in Morocco, published between January 1991 and February 2023, using Scopus, ScienceDirect, and PubMed databases. Medical Subject Heading keywords were used to guide the search.

**Results:**

This review included 18 studies focusing on different forms of extrapulmonary tuberculosis, such as cold abscesses, lymph nodes, meningeal, cutaneous, osteoarticular, genital, breast, and gastrointestinal tuberculosis. These studies provided insights into the clinical, diagnostic, and therapeutic aspects of these extrapulmonary tuberculosis forms. Prevalence data were limited, and treatment outcomes varied considerably across studies. The only study providing prevalence data for all forms of extrapulmonary tuberculosis reported a prevalence of 43.5% among tuberculosis cases in Larach Province. Treatment success rates ranged from 64.7% to 100%. Common risk factors included low socioeconomic status, diabetes mellitus, pulmonary tuberculosis exposure, and HIV co-infection.

**Conclusions:**

This review highlights a lack of comprehensive on the prevalence of extrapulmonary tuberculosis in Morocco, with inconsistent findings on risk factors and treatment outcomes. Further controlled studies are recommended to obtain more robust evidence and inform more effective management strategies for extrapulmonary tuberculosis.

## INTRODUCTION

Tuberculosis (TB) significantly affects global health and is a major cause of mortality among infectious diseases. Until the onset of the COVID-19 pandemic when it became the second leading cause of infectious mortality^1^, TB was the primary cause of deaths that were attributable to a single infectious agent, surpassing HIV/AIDS^1^. TB is primarily caused by *Mycobacterium tuberculosis* (Mtb), a member of the *Mycobacterium tuberculosis* complex, and is mainly transmitted through aerosols by individuals with active pulmonary disease^2^ ^,^ ^3^.

A 2016 global modelling study estimated that approximately 25% of the world’s population contracted Mtb^4^. It is estimated that in 2022, 10.6 million individuals developed active TB. Moreover, the number of TB-associated deaths increased between 2019 and 2021, reversing the downward trend recorded from 2005 to 2019^1^. In 2022, approximately 1.3 million deaths, including HIV-negative (1.13 million) and HIV-positive (167, 000) individuals, are linked to TB^1^. This represents a decrease from the estimates of 1.6 million in 2021^1^.

Although TB has traditionally been considered a pulmonary disease, recent studies have indicated that Mtb can disseminate from its initial infection site through various pathways and that it can potentially affect almost all human organs^5^. Pulmonary TB (PTB) diagnosis is easier because pathological samples are rich in Mtb. However, the diagnosis of extrapulmonary TB (EPTB) is challenging because non-respiratory pathological samples are generally poor for Mtb. Furthermore, these samples can be difficult to obtain because they may require invasive procedures such as bone biopsy, pleural biopsy, or cerebrospinal fluid analysis^6^.

EPTB is a highly diverse group of pathologies, with lymph node and pleural forms being the most common, followed by the osteoarticular and urogenital forms^7^ ^,^ ^8^. The incidence of EPTB has increased in recent years, whereas that of PTB has been declining^9^ ^,^ ^10^.

Global initiatives to stop the progression of TB include research across diverse domains, such as epidemiology, risk factors, immune response, TB pathophysiology, and the development of novel diagnostic and treatment methods for all forms of infection, including the disease itself^11^.

TB remains an important public health concern in Morocco. According to the 2022 estimates by the World Health Organization (WHO), approximately 35,000 individuals are affected by TB (estimated incidence rate: 93 cases per 100,000 population), and 47% of these cases are EPTB. It is responsible for 2,773 deaths, including HIV-negative (2,700) and HIV-positive individuals (73), with 180 patients developing multidrug-resistant TB^1^. In the same year, Morocco’s National TB Program (NTP) reported 29,327 new TB cases (incidence rate: 80 cases per 100,000 population), with 295 patients developing multidrug-resistant TB^12^. Analysis of data collected by the NTP showed that the distribution of TB cases by type changed significantly between 1980 and 2015. Indeed, among newly reported TB cases, the proportion of PTB decreased from 63% to 52%, whereas that of EPTB increased from 23% to 46%^13^.

To address this public health challenge, Morocco implemented the Moroccan National Strategic Plan (2021-2023), which extends the goals of the previous national strategic plan (2018-2021), in line with recommendations by the WHO. The plan aims to reduce TB-related mortality by 60% in 2023 compared to 2015 by enhancing the detection of new cases and improving treatment and patient monitoring^14^. By aligning its efforts with the strategies of the WHO, including the Directly Observed Treatment Strategy (1991), Stop TB (2006), and End TB (2016) strategies, Morocco has demonstrated a strong commitment to achieving Millennium Development Goals and, subsequently, sustainable development goals^14^.

This systematic review sought to pool findings from existing studies and address key questions regarding EPTB in Morocco, including its prevalence, risk factors, and treatment effectiveness. This comprehensive evaluation provides valuable insights for stakeholders including policymakers, healthcare professionals, and researchers to develop targeted strategies for high-risk populations.

## METHODS

### Literature search strategy and data sources

This systematic review adhered to the 2020 Preferred Reporting Items for Systematic Reviews and Meta-Analyses (PRISMA) guidelines^15^. The search, which was limited to articles published between January 1991 and February 2023, was performed on PubMed, Web of Science, and Scopus databases using MeSH descriptors. “Tuberculosis” AND “Morocco” were used as search terms.

### Inclusion and exclusion criteria

Our research mainly focused on observational studies involving EPTB in Morocco and included studies published between January 1991 and February 2023, which were mainly case series and cross-sectional studies. Non-English or French articles, studies conducted outside Morocco, articles without full access, studies involving PTB or PTB and EPTB, animal studies, conference abstracts, case reports, and duplicate articles were excluded.

### Data extraction and quality assessment

All references retrieved through the searches were imported into ZOTERO reference management software, which was first used to remove duplicates and then to screen article titles and abstracts. Studies meeting the eligibility criteria were selected for a thorough full-text review.

Next, the reviewers (MD and SB) individually assessed the full texts of the eligible articles and resolved any disagreements through discussion and confirmation by a third author, KH. Data including the first author’s name, year of publication, study location, study design, form of EPTB, sample size, sampling technique, average participant age, number of female participants, study period, diagnostic method, and key findings from each study were extracted collaboratively by MD and SB. The quality of the included cross-sectional studies was assessed using the Joanna Briggs Institute (JBI) critical appraisal tools for analytical cross-sectional studies^16^ (Table S1). Case series were assessed using the JBI critical appraisal checklist for case series^17^ (Table S2), and disagreements were resolved through discussion and consensus building.

**TABLE S1: Joanna:** Briggs Institute Critical Appraisal tool for the one cross-sectional study included in the conducted systematic review.

Included Studies	JBI quality assessment criteria’s								Total Score %
	Q1	Q2	Q3	Q4	Q5	Q6	Q7	Q8	
Bennani et al. (2019)	Y	Y	Y	Y	Y	Y	N	Y	87.5

**Note: Y:** Yes**; N:** No.

Q1: Were the criteria for inclusion in the sample clearly defined?

Q2: Were the study subjects and the setting described in detail?

Q3: Was the exposure measured in a valid and reliable way?

Q4: Were objective, standard criteria used for measurement of the condition?

Q5: Were confounding factors identified?

Q6: Were strategies to deal with confounding factors stated?

Q7: Were the outcomes measured in a valid and reliable way?

Q8: Was appropriate statistical analysis used?

**TABLE S2:** Quality assessment of the included case series using the Joanna Briggs Institute (JBI) Critical Appraisal Checklist for Case Series.

Study	Q1	Q2	Q3	Q4	Q5	Q6	Q7	Q8	Q9	Q10	Score
Aboulfalah et al. (2012)	Y	Y	Y	N	N	Y	Y	Y	Y	U	70%
Ahizoune et al. (2022)	Y	Y	Y	Y	Y	Y	Y	Y	N	Y	90%
Akhdari et al. (2006)	Y	Y	Y	Y	Y	N	Y	Y	N	U	70%
BayBay et al. (2021)	Y	Y	Y	Y	Y	Y	Y	Y	N	Y	90%
Benjelloun et al. (2015)	Y	Y	Y	N	N	Y	Y	Y	N	U	60%
Bouziyane et al. (2020)	Y	Y	Y	N	N	Y	Y	Y	Y	U	70%
Dollo et al. (2017)	Y	Y	Y	N	N	Y	Y	Y	N	Y	70%
El abkari et al. (2006)	Y	Y	Y	Y	Y	Y	Y	Y	N	Y	90%
Fedoul et al. (2011)	Y	Y	Y	N	N	Y	Y	Y	N	U	60%
Hamzaoui et al. (2014)	Y	Y	Y	N	N	Y	Y	Y	N	U	60%
kabiri et al. (2020)	Y	Y	Y	N	N	Y	Y	Y	N	U	60%
Samlani et al. (2011)	Y	Y	Y	Y	Y	Y	Y	Y	Y	U	90%
Teklali et al. (2003)	Y	Y	Y	Y	Y	Y	Y	Y	Y	U	90%
Zouhair et al. (2007)	Y	Y	Y	Y	Y	Y	Y	Y	N	U	80%

**Note: Y:** Yes**; N:** No. **U:** Unclear

Q1: Were there clear criteria for inclusion in the case series?

Q2: Was the condition measured in a standard, reliable way for all participants included in the case series?

Q3: Were valid methods used for identification of the condition for all participants included in the case series?

Q4: Did the case series have consecutive inclusion of participants?

Q5: Did the case series have complete inclusion of participants?

Q6: Was there clear reporting of the demographics of the participants in the study?

Q7: Was there clear reporting of clinical information of the participants?

Q8: Were the outcomes or follow up results of cases clearly reported?

Q9: Was there clear reporting of the presenting site(s)/clinic(s) demographic information?

Q10: Was statistical analysis appropriate?

### Data synthesis

A summary of the information extracted from the included studies is presented in Table 1. The findings were then compared and presented.

**TABLE 1:** Included studies and their Characteristics.

Author(s) (publication date)	Province	Study design	EPTB site	sample size	Sampling technique	Mean age (years)	Females	Study period	Diagnosis method	Study results	Quality score
Aboulfalah et al. (2012)	Marrakech	Retrospective (CS)	Genital	28	Medical records	33	28	2003-2009	Histological	The importance of identifying and diagnosing female genital TB as a potential cause of infertility in women, especially in resource-limited settings	70%
Ahizoune et al. (2022)	Rabat	Retrospective (CS)	Meningeal	40	Medical records	44 ±18 (>18)	15	2000-2017	Clinical, biological, and radiological	Clinical and epidemiological aspects of meningeal TB.	90%
										Febrile confusion was the most commonly reported manifestation among the study patients, followed by febrile meningeal syndrome.	
										The percentage of meningeal TB cases with favourable outcomes was 45%.	
Akhdari et al (2006)	Casablanca	Retrospective (CS)	Cutaneous	30	Medical records	11 (≤15)	14	1981-2004	Clinical, immunological, bacteriological, and histological	The epidemiological characteristics of cutaneous TB in children: Scrofuloderma and gumma were the most frequent forms.	70%
										Treatment outcomes: 73.33% patients had a favourable outcome with complete healing of lesions.	
BayBay et al. (2021)	Fez	Retrospective and prospective (CS)	Cutaneous	16	Medical records	10.5 (1.3-16)	9	2006-2017	Clinical, immunological, bacteriological, and histological	Cutaneous TB in children is dominated by gumma and scrofuloderma lesions.	90%
										Out of 147 cutaneous TB cases, 16 (10%) were children.	
										Treatment outcomes: 94% patients had a favourable outcome with complete healing of lesions.	
Benjelloun et al. (2015)	Rabat	Retrospective (CS)	Lymph node	30	Medical records	26 (10-69)	15	Not reported	Histological	The epidemiological, diagnostic, and therapeutic profile of lymph node TB; 93% patients had a favourable outcome.	60%
Bouziyane et al. (2020)	Casablanca	Retrospective (CS)	Breast	17	Medical records	33.5 (18-54)	17	2017-2019	Clinical, bacteriological, and histological	The epidemiological, diagnostic, and therapeutic aspects of breast TB: Breast TB is infrequently observed, primarily occurring during the period of genital activity.	70%
										The frequency of breast tuberculosis was 0.64% of all mastopathies.	
Dendane et al. (2013)	Rabat	Retrospective	Meningeal	508	Medical records	21-51	227	1999-2007	Bacteriological and/or clinical	A scoring-based diagnostic approach to distinguish tuberculous meningitis from bacterial meningitis, incorporating various clinical and laboratory criteria.	NA
Dollo et al. (2017)	Casablanca	Retrospective	Meningeal	52	Medical records	32 ±14 (14-77)	26	2011-2014	Direct examination and/or culture and/or PCR	Clinical and epidemiological aspects of meningeal TB.	70%
										Meningeal tuberculosis accounts for 10% of meningitis cases.	
El abkari et al (2006)	Fez	Retrospective (CS)	Peritoneal	123	Medical records	28 (2-66)	89	2001-2003	Mainly histological	The clinical characteristics, diagnostic challenges, and treatment outcomes of peritoneal TB in the study population; outcome was favourable in 90%.	90%
Fedoul et al. (2011)	Fez	Retrospective (CS)	Spinal	82	Medicals records	43.1(3.5-75)	44	2002-2006	Clinical, biological, histological, and radiological	Epidemiological, diagnostic, and therapeutic aspects of spinal TB localization	60%
Hamzaoui et al. (2014)	Marrakech	Retrospective (CS)	Lymph node	357	Medical records	29.1 (2 months - 88years)	223	2011-2012	Bacteriological and/or histological	The epidemiological, diagnostic, and therapeutic profile of lymph node TB; outcome was favourable in 95,2%	60%
Kabiri et al. (2020)	Rabat	Retrospective (CS)	Cold abscess	16	Medical records	39.1 (18-73)	4	2011-2017	Bacteriological and/or histological	Clinical and surgical management of chest wall cold abscesses caused by tuberculosis. Early diagnosis and treatment can lead to favourable outcomes in patients, with a return to normalcy following appropriate anti-TB treatment	60%
Lekhbal et al. (2020)	Casablanca	Retrospective	Lymph node	104	Medical records	24 (3-72)	69	May 2017-November 2018	Bacteriological and/or histological	Focused on lymph node TB treatment and identified lymph node size, disease recurrence, the existence of abscesses and fistulas, resistance to medical treatment, and the development of paradoxical reactions as significant indicators necessitating surgical intervention	NA
Samlani et al. (2011)	Marrakech	Retrospective (CS)	Abdominal	120	Medical records	38.3 (12-90)	75	2001-2009	Histological	Epidemiological, diagnostic, and therapeutic aspects of abdominal TB. Peritoneal TB was the most common location, followed by intestinal location; outcome was favourable in 95%	90%
Sbayi et al. (2020)	Larache	Retrospective	EPTB	2962	Medical records	31.74±18.83	1380	2000-2012	Not reported	The importance of considering age and gender, which appear to influence disease localization and treatment outcomes, in EPTB assessment and management	NA
										EPTB represents 43.5% of all tuberculosis patients in Larache province.	
Teklali et al. (2003)	Rabat	Retrospective (CS)	Bone	106	Medical records	8 (18 months-16years)	51	1980-2001	Epidemiological, clinical, biological, bacteriological, and histological	Focused on bone TB in children, excluding vertebral TB, with a different clinical presentation and generally positive outcomes following treatment	90%
Zouhair et al. (2007)	Casablanca	Retrospective (CS)	Cutaneous	216	Medical records	29 (4-90)	106	1981-2004	Bacteriological and/or histological	Epidemiological, clinical, histopathological, bacteriological, and therapeutical aspects of cutaneous TB; outcome was favourable in 93%	80%
Bennani et al. (2019)	Rabat, Casablanca and Fez	Cross-sectional	Lymph node	262	Is calculated based on prevalence in the 3 regions (questionnaire)	25 (all ages)	151	November 2016-May 2017	Histopathological, bacteriological, and GeneXpert	It is advisable to continue using histopathology to diagnose lymph node TB and to explore alternative techniques for enhancing diagnostic accuracy.	87.5%

**CS:** case series, **EPTB:** extrapulmonary tuberculosis, **NA:** not applicable, **TB:** tuberculosis.

### Ethics statement

This study did not require ethical approval or informed consent from the participants, because it examined published data and did not involve human or animal subjects.

## RESULTS

### Literature search and selection of eligible articles

**FIGURE 1:**
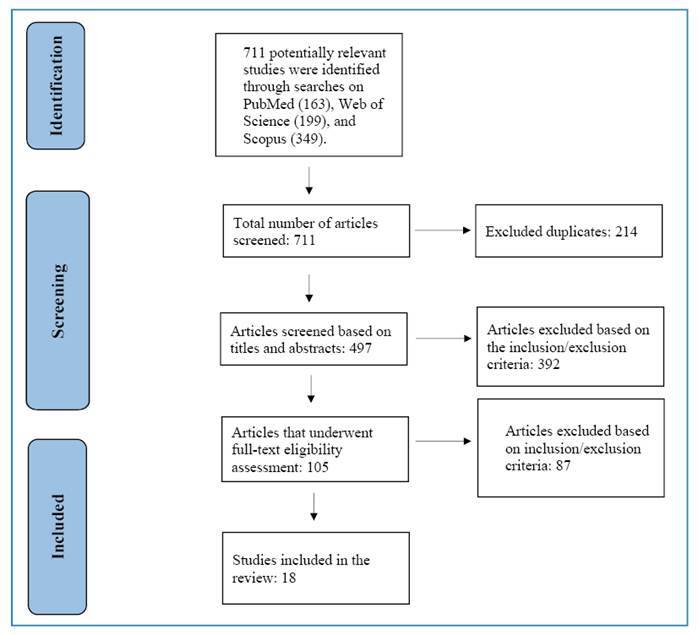
A PRISMA flow diagram of the study selection process.

The literature search retrieved 711 studies. After removing 214 duplicate articles, an initial screening of titles and abstracts led to the exclusion of an additional 392 studies, leaving 105 articles that met the criteria for a comprehensive full-text review and data evaluation. Ultimately, this systematic review involved 18 studies that met the eligibility criteria (14 case series studies, three retrospective studies, and one cross-sectional study) (Figure 1). The features of the included studies are listed in Table 1. A total of 18 studies had a sample size of 5,069.

### Results description

Eighteen studies reported the following forms of EPTB: lymph node TB (four)^18^ ^-^ ^21^, meningeal TB (three)^22^ ^-^ ^24^, cutaneous TB (three)^25^ ^-^ ^27^, osteoarticular TB (two)^28^ ^,^ ^29^, genital TB (one)^30^, breast TB (one)^31^, abdominal TB (two)^32^ ^,^ ^33^, tuberculous cold abscess (one)^34^, and EPTB in a broad context (one)^35^.

### Prevalence of EPTB

The prevalence of EPTB in Morocco has varied across studies. While some studies did not provide data on the prevalence of specific EPTB forms^18^ ^,^ ^20^ ^-^ ^22^ ^,^ ^24^ ^,^ ^29^ ^,^ ^30^ ^,^ ^32^ ^,^ ^34^, one study reported cutaneous TB in 14% of 216 TB cases^25^, and another noted a 1.95% prevalence of cutaneous TB among all patients hospitalized in the dermatology department during the study period^27^. A study that assessed cutaneous tuberculosis in children found that out of 147 cutaneous TB cases, 16 (10%) were in children^26^. Breast TB accounted for 0.64% of mastopathies in one study involving women with breast disease^31^, while TB meningitis constituted 10% of 503 patients hospitalized for all forms of meningitis in another study^23^. 

One study reported that during the study period, peritoneal TB accounted for 6.1% of hospitalisations^33^, and another study reported that lymph node TB represented 20.8% of all EPTB forms and 38% of new EPTB cases^19^. Additionally, a study involving 106 cases of peripheral osteoarticular TB constituted 5.4% of hospitalizations over the last 20 years^28^. Furthermore, a study in the Larach Province reported that the prevalence of EPTB was 43.5% among tuberculosis cases^35^.

### Risk factors associated with EPTB

The majority of studies identified low socioeconomic status, diabetes mellitus, pulmonary TB exposure, and HIV as potential risk factors for EPTB. However, these studies were limited by the absence of statistically significant evidence of an association between these risk factors and EPTB.

### Treatment outcomes of EPTB

Most studies reported the percentage of patients with favorable treatment outcomes. Cutaneous TB has treatment success rates ranging from 73.33%^25^ to 94%^26^ ^,^ ^27^, while lymph node TB outcomes range from 93%^18^ to 95.2%^19^. Meningeal TB had fewer favorable outcomes, with rates ranging between 45%^22^ and 69%^23^. In previous studies, patients with osteoarticular TB had a recovery rate of 100% in the reported studies^23^ ^,^ ^28^. However, the favorable outcomes of other EPTB forms mentioned in this systematic review varied, ranging from 64.7% to 100%^31^ ^,^ ^32^ ^,^ ^34^. 

As most of the studies included in this review were case series, they focused on the clinical, diagnostic, and therapeutic aspects of various forms of EPTB. 

## DISCUSSION

This systematic review aimed to pool reported findings from existing studies and address questions regarding the prevalence, key risk factors, and treatment outcomes of EPTB. To the best of our knowledge, this is the first systematic review designed to identify, summarize, and quantify the available evidence to determine EPTB burden in Morocco. 

EPTB is rarely covered in public health literature. However, numerous clinical case reports and case series have been published describing patients with various types of EPTB. In these publications, EPTB was often regarded as a clinical anomaly rather than a public health concern^9^, which is also true for most publications in Morocco. One reason EPTB is likely not prioritized on the public health agenda is that it does not significantly contribute to disease transmission^9^.

This review highlighted the lack of research on the national prevalence studies. It should be noted that although numerous studies have reported the prevalence of some forms of this disease at the health facility level, or in some cases, the single-city level, a comprehensive national-level assessment is lacking.

Furthermore, this review revealed significant variability in the scope and focus of the included studies. The available literature primarily addresses specific forms of EPTB, such as meningeal, lymph node, and cutaneous tuberculosis, rather than providing a comprehensive overview of EPTB as a whole. This heterogeneity, which encompasses differences in sample sizes, study designs, diagnostic methods, and reported outcomes, presents challenges in synthesizing data for a cohesive understanding of EPTB prevalence, risk factors, and treatment results across the country.

Moreover, the variation in study quality and lack of data in some areas further complicate efforts to draw broad conclusions. Despite these limitations, the findings of these studies offer valuable insights into the epidemiological patterns and clinical outcomes associated with different forms of EPTB in Morocco.

This review highlights the significant variability in the reported prevalence of different forms of the disease in Morocco. For instance, the prevalence of cutaneous TB is relatively well documented, with one study reporting a prevalence of 1.95% among all patients hospitalized in the dermatology department during the study period^27^. This rate is higher than that reported in a study conducted in India^36^, where only 0.1% of patients with dermatological disorders had cutaneous tuberculosis; this percentage is even lower in developed countries (0,066%)^37^. In addition, 10% of the cutaneous TB cases were found in children, highlighting the impact across age groups^26^.

Breast TB, on the other hand, was less common, accounting for 0.64% of all mastopathies^31^, a rate higher than the 0.3% reported in South Africa^38^ but lower than the 3.4% observed in India^39^. 

Meningeal TB, reported to account for 10% of all meningitis cases^23^, was less common than that in a similar study conducted by Navarro-Flores et al., which found a prevalence of 14.63%^40^. Peritoneal TB accounted for 6.1% of hospitalizations^33^. Additionally, lymph node TB represented 20.8% of all EPTB forms and 38% of new EPTB cases^19^, consistent with the findings of a study conducted in Africa (24%)^41^ but lower than that in Brunei Darussalam (44.8%)^7^.

Furthermore, a study conducted in Larach Province reported that the prevalence of EPTB was 43.5%^35^, which is consistent with the findings of a study conducted in England and Wales (41%)^10^ but higher than that in Africa (26%)^41^. 

The variability in these prevalence rates suggests that, while certain forms of EPTB are relatively prevalent in Morocco, the overall burden of EPTB is challenging to quantify owing to differences in study design, population, and settings.

The studies included in this review identified several potential risk factors for EPTB, including low socioeconomic status, diabetes mellitus, pulmonary TB exposure, and HIV infection. These findings align with previous literature review^42^, which consistently highlighted these factors as being associated with EPTB. However, the absence of statistically significant evidence in many of these studies limits their ability to draw strong conclusions. This limitation underscores the need for more robust, controlled studies to establish a clearer association between these risk factors and EPTB.

The treatment outcomes for EPTB in Morocco vary across different forms of the disease. For example, cutaneous TB has a high treatment success rate of 94%^26^ ^,^ ^27^, while another study reported a lower success rate of 73.33%^25^. Lymph node TB showed favorable outcomes in 93%^18^ and 95.2%^19^ of the cases, respectively. However, meningeal TB outcomes were less favorable, with success rates of 45% in one study^22^ and 69% in another^23^. Osteoarticular TB has particularly favorable outcomes, with both studies reporting 100% recovery rates^28^ ^,^ ^29^.

These results are largely consistent with Morocco's national therapeutic success rates of 88% and 87%, as reported by the WHO (Global Tuberculosis Report 2023^1^) and Morocco’s NTP^12^, respectively. The findings across different studies and forms of EPTB indicate that while treatment outcomes are generally positive, there is significant variability depending on the specific form of EPTB and the context in which the treatment is administered.

This systematic review has some limitations that should be considered when interpreting the results. First, most of the included studies were case series, which are considered lower in the hierarchy of evidence because they have a limited ability to establish causation and are susceptible to selection bias. The lack of controlled studies limits our ability to draw strong conclusions about EPTB’s risk factors and treatment outcomes of EPTB in Morocco. Second, the studies included in this review involved different forms of EPTB and differed in various variables, including sample sizes, study populations, and diagnostic methods. This heterogeneity makes it difficult to perform a quantitative meta-analysis, estimate Morocco’s national EPTB prevalence, or generalize our findings to the entire population. 

Despite certain limitations, the findings of this review, combined with those of other studies, underscore the urgent need for further research on the prevalence, risk factors, and treatment outcomes of EPTB in Morocco. Comprehensive national surveys and methodologically robust studies are essential for a better understanding of the full scope of EPTB, which is crucial for developing effective public health strategies and improving clinical management. Socioeconomic factors have been identified as the key risk factors, highlighting the need for integrated social and healthcare interventions to improve health outcomes and reduce disease transmission. Historically underprioritized owing to its low contribution to transmission, EPTB requires increased focus through national TB control strategies that incorporate EPTB into policy, ensuring adequate resource allocation, research, and treatment efforts.

## CONCLUSION

This systematic review highlights the lack of comprehensive data on the prevalence of EPTB in Morocco as well as inconsistencies in treatment outcomes across studies. The included studies provided additional valuable information on the clinical and diagnostic aspects of various forms of EPTB. Common risk factors identified included low socioeconomic status, diabetes mellitus, pulmonary TB exposure, and HIV co-infection. The success rate of the treatment outcomes ranged from 64.7% to 100%. Further controlled studies are required to obtain more robust evidence.
